# Impact of the Promoting Physical Activity in Regional and Remote Cancer Survivors intervention on health-related quality of life in breast and colorectal cancer survivors

**DOI:** 10.3389/fonc.2024.1368119

**Published:** 2024-09-06

**Authors:** Sarah J. Hardcastle, Marta Leyton-Román, Chloe Maxwell-Smith, Dana Hince

**Affiliations:** ^1^ Sport and Physical Activity Research Centre, Sheffield Hallam University, Sheffield, United Kingdom; ^2^ Institute for Health Research, The University of Notre Dame, Fremantle, WA, Australia; ^3^ Department of Didactics of Musical, Plastic and Body Expression, University of Extremadura, Caceres, Spain; ^4^ School of Population Health, Curtin University, Perth, WA, Australia

**Keywords:** quality of life, exercise, health disparities, oncology, wearable technology

## Abstract

**Background:**

The PPARCS trial examined the efficacy of a distance-based wearable and health coaching intervention to increase physical activity (PA) in breast and colorectal cancer (CRC) survivors living in non-metropolitan areas. This paper examines the effects of the intervention on health-related quality of life (HRQoL) at 12 weeks (T2; end of intervention) and 24 weeks (T3; follow-up).

**Methods:**

Participants that were insufficiently physically active and had successfully completed cancer treatment were randomised to an intervention or control group. PA was assessed using an ActiGraph (GT9X) at baseline, T2, and T3. Intervention effects on HRQoL were analysed using quantile regression comparing treatment groups across time.

**Results:**

A total of 87 were randomised to intervention and control groups. There were generally no statistically significant differences between the groups on any HRQoL item except for pain. There was an arm (F(1, 219) = 5.0. p = 0.027) and time (F(2,221) = 4.8, p = 0.009) effect, reflecting the higher pain scores in the control group when collapsed across time points (median difference 16.7, CI 1.9 to 31.4, p = 0.027). For global HRQoL, the intervention group increased by 8.3 points between T1 and T2. The overall group median when collapsed across time was 16.7 points CI 8.2 to 25.2, p <0.001) greater in the intervention group than controls.

**Conclusions:**

While the PPARCS intervention resulted in significant increases in PA, participants indicated a high HRQoL at baseline, leaving little room for improvement. Findings suggest that PA may improve global HRQoL and pain in breast and CRC survivors.

## 1 Introduction

Physical activity (PA) is important for a healthy cancer survivorship trajectory. Being physically active post diagnosis is vital to reducing the risks of cancer-specific and all-cause mortality and improving survival (1, 2). Cancer survivors are also susceptible to a range of treatment-related side effects including fatigue, pain, insomnia, neuropathy, and lymphoedema (3) that may adversely affect quality of life (QoL) (4). PA has been found to improve health-related QoL (HRQoL) and reduce anxiety, fatigue, pain, and sleep problems in cancer survivors (5). Despite increasing evidence that PA improves cancer outcomes, many survivors do not meet the recommendations (6) to participate in at least 150 min of moderate-intensity aerobic PA per week and resistance or strength training at least twice weekly (7).

There are also substantial geographic inequalities related to health and survival. Cancer survivors who reside in non-metropolitan areas are more likely to be insufficiently active and obese (8, 9) and have poorer cancer survival compared with their counterparts living in major cities (8, 10). There is also evidence that non-metropolitan cancer survivors have a poorer QoL compared with their metropolitan counterparts (11, 12). Furthermore, non-metropolitan cancer survivors face barriers to PA engagement such as the availability of and access to exercise programs, alongside their cost (13). Effective, distance-based PA interventions have the potential to reduce health inequalities for non-metropolitan cancer survivors by reducing barriers to exercise and facilitating PA participation.

Distance-based interventions have demonstrated improvements in health outcomes including HRQoL and cancer-related symptoms (14, 15). The review of mobile health (mHealth) interventions on QoL in cancer survivors by Buneviciene et al. (2021) (15) found a statistically significant improvement in global Health Status (mean difference of 7.05 in PA interventions), similar to the effects (weighed mean difference of 6.78) of a meta-analysis of PA interventions on HRQoL in breast cancer survivors (16). However, only one (17) out of four RCTs (17–20) demonstrated superiority of the mHealth-delivered PA intervention on HRQoL over the control group. Furthermore, none of these interventions used smart wearables in conjunction with health coaching or recruited only non-metropolitan cancer survivors. In addition, only one study (21) was based on CRC and recruited patients during treatment. A gap in the literature exists on the effectiveness of wearable activity technology interventions to promote PA and preserve or improve HRQoL in geographically underserved breast and CRC survivors following treatment.

The Promoting Physical Activity in Regional and Remote Cancer Survivors (PPARCS) trial explored the efficacy of a smart wearable device (the Fitbit Charge 2™), in conjunction with telephone-health coaching in an entirely distance-based intervention to increase moderate-to-vigorous PA (MVPA) in Australian breast and colorectal cancer survivors (22). The PPARCS intervention significantly increased MVPA with a between-group net difference in MVPA of 50 min/week favouring the intervention group (23), which was maintained at follow-up (24). The improvement or preservation of HRQoL is important in cancer survivorship and is often included as a secondary outcome measure in clinical trials (25).

The primary aim of this paper was to report the effects of the PPARCS trial on HRQoL in non-metropolitan breast and CRC survivors. Secondary aims were to explore within group changes across time.

## 2 Methods

The trial was a two-arm multicentre randomised controlled trial (RCT) conducted across five Australian states (New South Wales, Victoria, Western Australia, South Australia, and Tasmania) from March 2019 through to February 2021. The study was approved by the St. John of God Hospital Human Research Ethics Committee (Reference #1201) and registered (ACTRN12618001743257). Written informed consent was obtained from participants prior to enrolment. The protocol and trial design have been described previously (22). An overview of the methods is outlined below.

### 2.1 Participants

Participants included adult breast cancer and CRC survivors who had completed active cancer treatment in the 5 years prior to recruitment. In brief, participants were recruited based on (a) remoteness and (b) low levels of PA. Remoteness was measured according to the accessibility/remoteness index of Australia and the Australian Statistical Geography Standard, which define five statistical areas: major cities, inner regional (IR), outer regional (OR), remote (R), and very remote (VR) (26). For international comparison, approximately 28% of Australians reside in regional and remote areas. Approximately 4,608,000 (17.9%) and 2,067,000 (8%) reside in inner and outer regional areas, respectively. A further 291,000 (1.1%) and 201,000 (0.8%) reside in remote and very remote areas (27). Eligible participants resided in non-metropolitan areas, were insufficiently physically active (i.e., engaging in <150 min of moderate-intensity PA per week), and had internet access via a computer or smartphone.

### 2.2 Recruitment

Eligible participants were identified from oncologists’ medical records and were mailed a participant information sheet and invitation letter from their treating oncologist. Individuals who expressed interest were screened by telephone to ensure eligibility using a screening questionnaire (which included the Active Australia Survey (28) to assess PA status) to determine eligibility. Written consent to participate was obtained following confirmation of eligibility.

### 2.3 Randomization

Following baseline assessments, an independent statistician, who was blinded to the assessments and intervention, randomised participants using consecutive randomisation codes (STATA Version 15; StataCorp., College Station, TX, USA) with a 1:1 allocation in block sizes of 4 and 6 to support allocation concealment. Participant allocation was implemented using sequentially numbered envelopes that were opaque and sealed. Following consent and baseline assessment, the trial coordinator opened the next envelope in the sequence and wrote the participant study number onto it prior to allocating the participant to that group. If the participant was allocated to the intervention group, the trial coordinator mailed out a Fitbit Charge 2™ (Fitbit LLC, San Francisco, CA, USA) along with detailed instructions on tracker set-up and functions.

### 2.4 Design

#### 2.4.1 Intervention arm

The 12-week intervention was designed by the first author and consisted of two components, which have been described previously (22):

1. Smart tracker: The Fitbit Charge 2™ (Fitbit LLC, San Francisco, CA, USA) is a wrist-worn device that displays steps, distance, heart rate, and active minutes while providing automated prompts to nudge participants to accumulate at least 250 steps/h. Data from the device were uploaded to the Fitbit app via Bluetooth.
2. Health coaching: The purpose of the telephone health coaching was to motivate increased PA and reduced sedentary behaviour by supporting participants’ self-efficacy, action planning, and problem solving. The first session (week 1; up to 60 min) covered technical issues and features of the Fitbit device and sought to foster positive outcome expectancies and confidence towards PA by emphasising the importance of MVPA and providing information on the risks of inactivity, and by guiding participants to create an action plan for their PA engagement over the following week. Three follow-up sessions (weeks 2, 4, and 8; approximately 30 min each) provided feedback on PA behaviour, assistance with problem solving, and support with updating goals and action plans as participants progressed. A patient-centred and stepped-care approach was adopted by providing additional health-coaching sessions (i.e., at weeks 6 and 10) to those who needed them to achieve meaningful sustained PA change. The intervention did not prescribe exercise but rather focused on increasing time spent in MVPA to achieve the PA recommendations (7), and this was individually tailored according to baseline PA and motivation. The optimal exercise target was at least 180 min of moderate-intensity PA, based on research demonstrating better long-term survival in cancer survivors who engaged in 3–5 h of moderate-intensity PA per week (29).

#### 2.4.2 Control arm

This group received a mailed booklet (which was also given to the intervention group) designed to educate and motivate improvements in PA. The booklet provided, “Exercise for People Living with Cancer” (2016 edition, reprinted 2017; https://www.cancer.org.au), is freely available from Cancer Council Australia and widely distributed, represented usual care.

Following the end of intervention (12 weeks), participants commenced a 12-week maintenance period. Intervention participants kept their Fitbit Charge 2™ but received no further intervention or support. The control group did not receive any further support.

#### 2.4.3 Data collection

Data collection was conducted remotely at baseline (T1) and week 12 (T2) and week 24 (T3). Once eligibility was confirmed, participants were mailed the study questionnaire, an ActiGraph GTX9 accelerometer (ActiGraph, Pensacola, FL, USA), written accelerometer instructions (e.g., worn on the right hip for 7 consecutive days for all waking hours), and a reply-paid satchel. ActiGraph GTX9 data were processed using 60-s epochs, using the ActiLife software package. Freedson cut points of ≥1952 counts per minute (cpm) were used to quantify MVPA (30). A cut point of <100 cpm denoted sedentary behaviour.

HRQoL was measured at the three time points using the European Organisation for Research and Treatment of Cancer QoL questionnaire (EORTC QLQ-C30, version 3) (31), which has been widely used to assess QoL in cancer survivors (32). The EORTC QLQ-C30 is a 30-item measure of HRQoL consisting of five multi-item functional scales (physical, role, emotional, cognitive, social), three multi-item symptom scales (fatigue, pain, nausea, and vomiting), six single-item symptom scales (dyspnoea, insomnia, appetite loss, constipation, diarrhoea, and financial difficulties), and one multi-item global QoL scale. Each item has a four-point response scale from “not at all” to “very much,” and scores are calculated using a linear conversion to create a score from 0 to 100 with a higher score signifying a higher QoL.

### 2.5 Statistical methods

EORTC QLQ-C30 scale distributions were highly skewed and as such were summarised with the medians and interquartile range (IQR). Comparison of the groups over time was conducted for all EORTC scales using regression models including arm (control v intervention), time (baseline, 12 weeks, and 24 weeks), and the interaction between these two as fixed effects. For multi-item scales, quantile regression was used to model the median response as a linear function of the dependent variables. Standard error estimates for these models were calculated after allowing for clustering within participants using the qreg2 (33) package (StataCorp v18, LLC, Texas, USA). The six single-item symptom scales (dyspnoea, insomnia, appetite loss, constipation, diarrhoea, and financial difficulties) have only four possible values in their distributions, some of which were not present in all combinations of arm and time. These variables were therefore dichotomised as “not at all” or “all other responses” and analysed using a logistic regression model with a random effect for participant to account for the correlation in the data. An intervention effect on HRQoL scales was inferred if the interaction returned p<0.05.

## 3 Results

**Figure 1:**
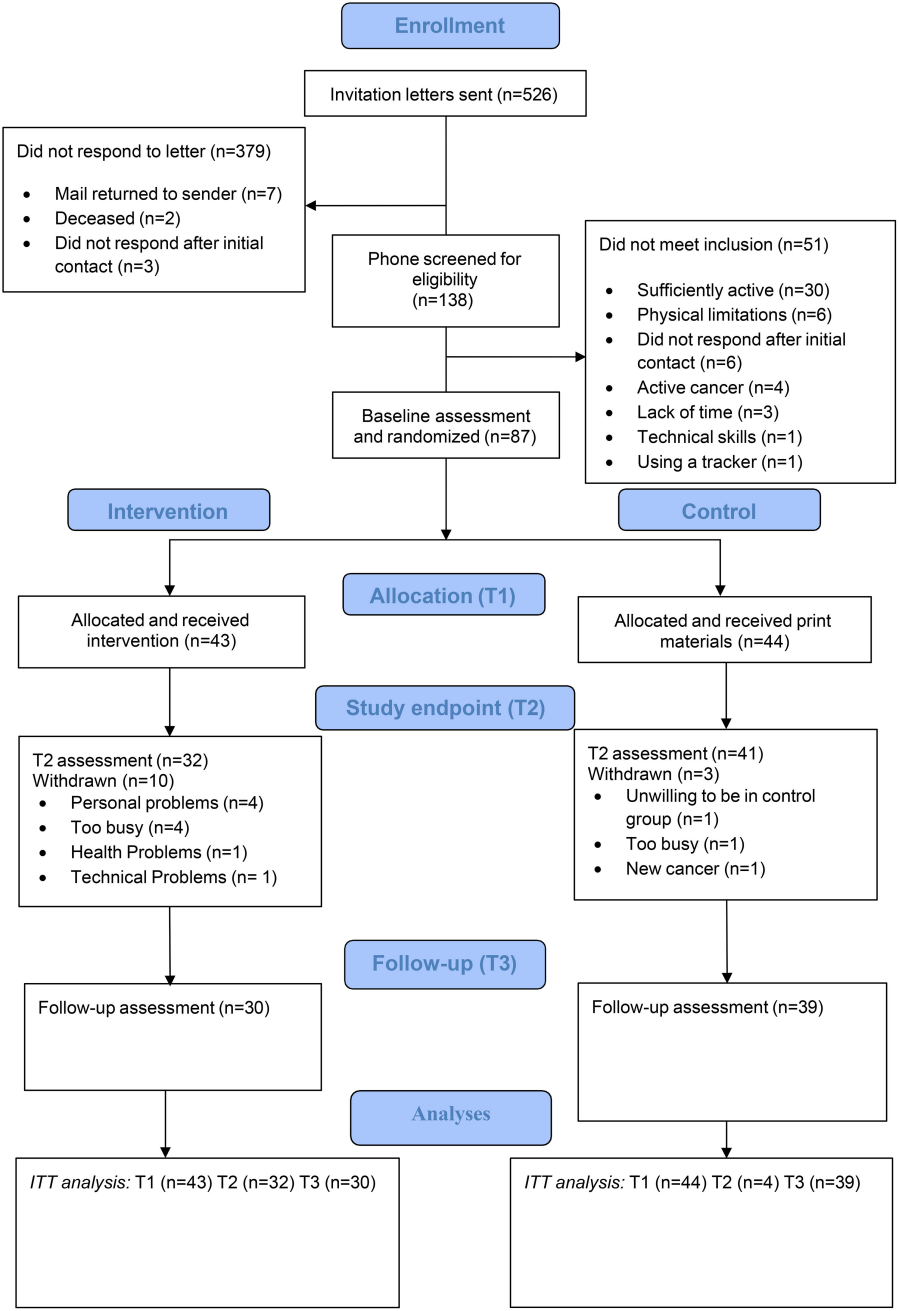
CONSORT diagram of PPARCS trial.

A total of 87 participants were randomised to intervention (*n* = 43) and control (*n* = 44) groups. **Figure 1** displays the reach, enrolment, and allocation of participants, and **Supplementary File 1** includes the Consolidated Standards of Reporting Trials (CONSORT) checklist for the reporting of the study (34). Demographic characteristics were similar across groups at baseline (**Table 1**). There were 69 participants (79%) who remained in the trial at T3 (72.1% in the intervention group). Those who remained at T3 did not differ from those who did not by age, gender, baseline MVPA, cancer type, or months since diagnosis. The medians (IQR) in both arms across the duration of the study are presented in **Table 2** for all scales comprising the EORTC QLQ-C30.

**Table 1:** Baseline characteristics of participants.

	Overall (*n* = 87)	Intervention (*n* = 43)	Control (*n* = 44)
Age (year)	63.1 ± 11.1	63.7 ± 10.1	62.6 ± 11.8
Gender (female)	74 (85.1%)	38 (88.4%)	36 (81.8%)
Marital status			
Married/in a relationship	64 (73.6%)	32 (74.4%)	32 (72.7%)
Divorced/separated	9 (10.4%)	6 (13.9%)	3 (6.8%)
Single	7 (8.0%)	0 (0.0%)	7 (15.9%)
Widowed	7 (8.0%)	5 (5.7%)	2 (5.6%)
Education			
University degree	26 (30%)	14 (33%)	12 (27%)
Post-school training	29 (33%)	13 (30%)	16 (36%)
High school	28 (32%)	13 (30%)	15 (34%)
Other/no qualifications	4 (5%)	3 (7%)	1 (3%)
Household income (AUD)			
≤30,000	19 (22%)	12 (28%)	7 (16%)
30,001–52,000	20 (23%)	5 (12%)	15 (34%)
52,001–104,000	23 (26%)	11 (26%)	12 (27%)
104,001–156,000	11 (13%)	6 (14%)	5 (11%)
≥156,000	8 (19%)	4 (9%)	5 (9%)
Missing	6 (7%)	5 (11%)	1 (3%)
Australian state			
New South Wales	53 (61.0%)	28 (65.1%)	25 (56.8%)
Victoria	17 (19.5%)	8 (18.6%)	9 (20.4%)
Western Australia	14 (16.1%)	6 (14.0%)	8 (18.2%)
South Australia	2 (2.3%)	1 (2.3%)	1 (2.3%)
Tasmania	1 (1.1%)	0 (0.0%)	1 (2.3%)
Cancer type			
Breast	66 (75.9%)	34 (79.1%)	32 (72.7%)
Colorectal	21 (24.1%)	9 (20.9%)	12 (27.3%)
Comorbidity score	6.5 ± 5.1	6.5 ± 4.9	6.5 ± 5.4
Months since diagnosis^a^	24.8 ± 12.7	25.1 ± 12.4	24.5 ± 13.1
Treatment			
Surgery only	13 (15.0%)	8 (19.0%)	5 (11.0%)
Surgery with chemotherapy	47 (54.0%)	19 (44.0%)	28 (64.0%)
Surgery with radiation therapy	53 (60.9%)	26 (59.1%)	27 (62.8%)
Hormone therapy	31 (35.6%)	17 (38.6%)	14 (32.6%)
Remoteness classification			
Inner regional	32 (36.9%)	16 (37.2%)	16 (36.4%)
Outer regional	51 (58.6%)	25 (58.2%)	26 (59.1%)
Remote	3 (3.4%)	1 (2.3%)	2 (4.5%)
Very remote	1 (1.1%)	1 (2.3%)	0 (0.0%)

a Months since diagnosis (mean ± SD) was available for Control (n = 30) and Intervention (n = 29) participants. Data are presented as mean ± SD or n (%). Abbreviations: AUD, Australian dollar.

**Table 2:** Median EORTC QLQ-C30 scale scores for baseline, end of intervention (12 weeks), and post intervention follow-up (24 weeks).

EORTC QLQ-C30 v 3.0	Baseline		12-week		24-week	
	Control (n = 44)	Intervention (n = 43)	Control (n = 41)	Intervention (n = 32)	Control (n = 38)	Intervention (n = 31)
	Median (IQR)	Median (IQR)	Median (IQR)	Median (IQR)	Median (IQR)	Median (IQR)
QoL Global health status	66.7 58.3 to 83.3	75.0 58.3 to 83.3	66.7 66.7 to 83.3	83.3 66.7 to 83.3	66.7 58.3 to 83.3	83.3 66.7 to 91.7
Functional scales						
Physical functioning	93.3 80.0 to 93.3	93.3 86.7 to 100.0	86.7 80.0 to 93.3	93.3 80.0 to 100.0	86.7 73.3 to 93.3	93.3 80.0 to 100.0
Role functioning	100.0 66.7 to 100.0	100.0 83.3 to 100.0	100.0 83.3 to 100.0	100.0 66.7 to 100.0	100.0 66.7 to 100.0	100.0 66.7 to 100.0
Emotional functioning	75.0 66.7 to 91.7	83.3 66.7 to 100.0	75.0 58.3 to 91.7	83.3 66.7 to 100.0	75.0 66.7 to 91.7	75.0 66.7 to 91.7
Cognitive functioning	83.3 83.3 to 100.0	83.3 83.3 to 100.0	83.3 66.7 to 100.0	83.3 83.3 to 100.0	83.3 66.7 to 100.0	100.0 83.3 to 100.0
Social functioning	100.0 66.7 to 100.0	100.0 66.7 to 100.0	83.3 66.7 to 100.0	100.0 83.3 to 100.0	100.0 66.7 to 100.0	100.0 83.3 to 100.0
Symptom scales/items						
Fatigue	22.2 11.1 to 44.4	22.2 11.1 to 44.4	22.2 11.1 to 33.3	22.2 11.1 to 33.3	22.2 11.1 to 33.3	22.2 11.1 to 33.3
Nausea and vomiting	0.0 0.0 to 0.0	0.0 0.0 to 0.0	0.0 0.0 to 0.0	0.0 0.0 to 0.0	0.0 0.0 to 0.0	0.0 0.0 to 0.0
Pain	16.7 0.0 to 33.3	0.0 0.0 to 33.3	16.7 0.0 to 33.3	0.0 0.0 to 33.3	33.3 0.0 to 33.3	16.7 0.0 to 33.3
Dyspnoea	0.0 0.0 to 33.3	0.0 0.0 to 33.3	0.0 0.0 to 33.3	0.0 0.0 to 33.3	0.0 0.0 to 33.3	0.0 0.0 to 0.0
Insomnia	33.3 16.7 to 66.7	33.3 0.0 to 66.7	33.3 0.0 to 33.3	33.3 0.0 to 33.3	33.3^3^ 0.0 to 66.7	33.3 33.3 to 33.3
Appetite loss	0.0 0.0 to 0.0	0.0 0.0 to 33.3	0.0 0.0 to 0.0	0.0 0.0 to 0.0	0.0 0.0 to 0.0	0.0 0.0 to 0.0
Constipation	0.0 0.0 to 0.0	0.0 0.0 to 33.3	0.0 0.0 to 33.3	0.0 0.0 to 0.0	0.0 0.0 to 0.0	0.0 0.0 to 33.3
Diarrhoea	0.0 0.0 to 0.0	0.0 0.0 to 0.0	0.0 0.0 to 0.0	0.0 0.0 to 0.0	0.0 0.0 to 0.0	0.0 0.0 to 33.3
Financial difficulties	0.0 0.0 to 33.3	0.0 0.0 to 33.3	0.0 0.0 to 33.3	0.0 0.0 to 33.3	0.0 0.0 to 33.3	0.0 0.0 to 0.0

IQR, interquartile range. Missing data: Baseline Control—Global health (n = 1); 12 weeks Control—Global health (n = 4); Physical and role function, Fatigue, Pain, and Dyspnoea (n = 2), all others (n = 3); 12-week Wearables—All scales (n = 1) except Insomnia (n = 2); 24-weeks Control—Global health, Physical, Emotional, Cognitive and Social function, Fatigue, Nausea and vomiting, Pain, Insomnia and Appetite loss (n = 1), all others (n = 2); 24-weeks Wearables—Global health, Role function, and Dyspnoea (n = 1).

### 3.1 Global quality of life

The median global QoL score for the control group remained at 67 (IQR 58 to 83) points across all time points. The intervention group median was higher than controls from baseline and then increased further to 83 at 12 weeks and at follow-up (see **Table 2**). There was no statistical evidence for this differential pattern over time when compared with the control group (Arm by Time interaction: F(2, 215) = 0.4, p = 0.701). The overall group median when collapsed across time, however, was 16.7 points (95% CI 8.2 to 25.2, p <0.001) greater in the intervention than the control group.

### 3.2 Functional scales

Role and social and cognitive function scale scores demonstrated significant ceiling effects, with the median score in both groups at baseline (and indeed across all time points) at least 83.3, which is the second highest value the transformed score can take (see **Table 2**). Not surprisingly, the median regression models failed to converge, likely due to the lack of variability in the observed data. Physical and social function scores were also highly skewed towards the upper (high functioning) end of the scale at baseline, T2, and T3, but there was slightly more variability in the medians between groups and time points and the values were lower at baseline than the other function scales (see **Table 2**). There was no evidence for an arm by time interaction for Physical (F(2, 219) = 1.3, p = 0.263) or Social (F(2, 218) = 1.0, p = 0.357) function, nor any between group effects on these two scales (Physical function median difference 6.7, 95% CI −4.8 to 18.1, p = 0.252; Emotional function median difference 8.3, 95% CI −1.6 to 18.3, p = 0.100).

### 3.3 Multi-item and single symptom scales

The multiple-item symptoms scales had response patterns also suggestive of significant floor effects (see **Table 2**). There was no evidence for an interaction between arm and time on the median values for these outcomes (Fatigue F(2, 219) = 0.0, p = 1.000; Nausea and Vomiting failed to converge; Pain F(2, 219) = 0.0, p = 1.000). There was an effect of arm (F(1, 219) = 5.0. p = 0.027) and time (F(2,221) = 4.8, p = 0.009), reflecting the higher median pain scores in the Control group when collapsed across time points (median difference 16.7, 95% CI 1.9 to 31.4, p = 0.027), and the increase in median Pain QoL scores between baseline and 24 weeks in both groups across the course of the study (median difference 16.6, 95% CI 4.8 to 28.5, p = 0.006; see **Table 2**).

**Figure 2:**
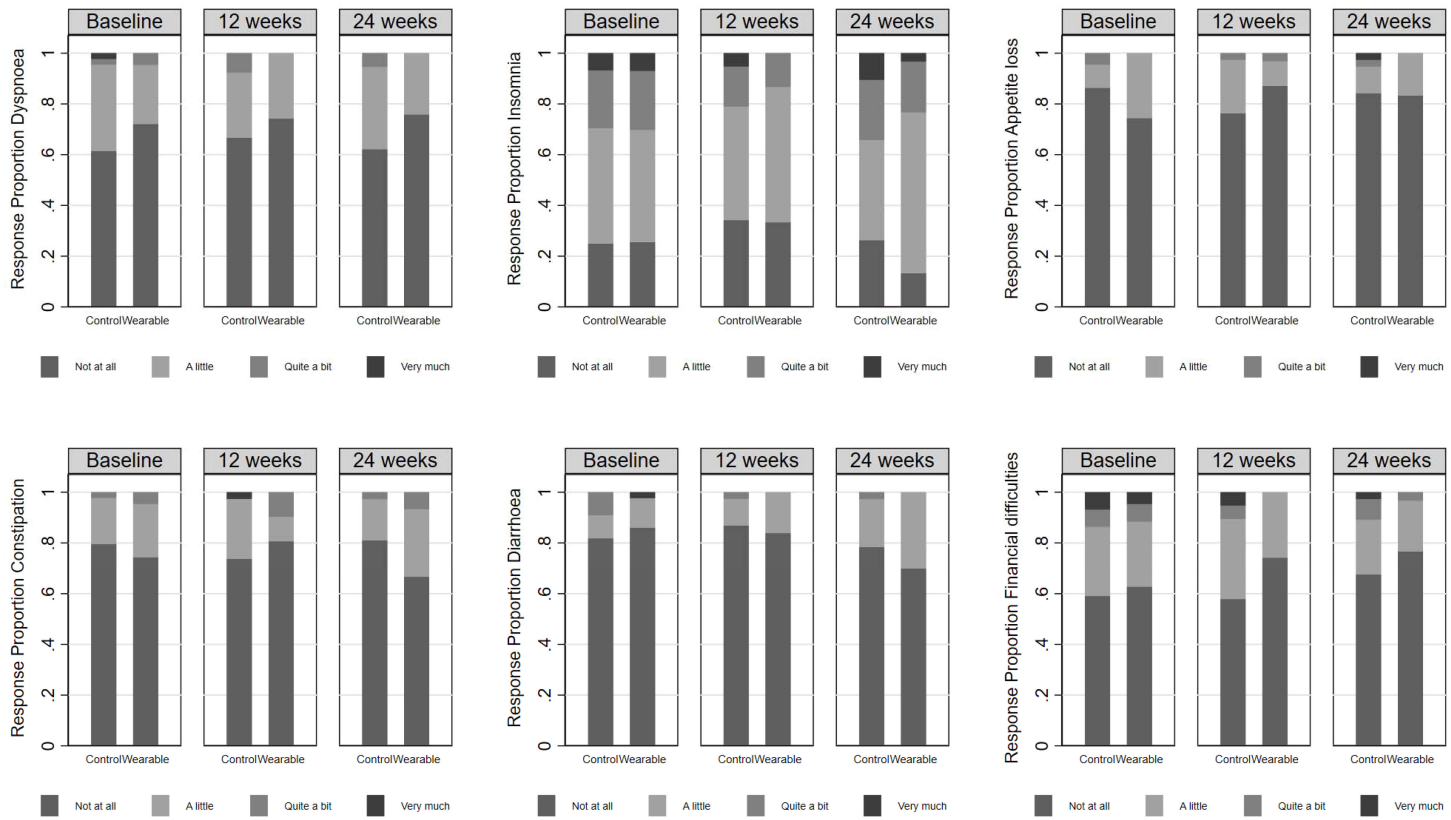
The proportion of participants responding to each of the response category options for each single item scale in the EORTC QLQ-C30 v 3.0 as a function of arm and time. The very high proportion of individuals responding “Not at all” at baseline indicates substantial floor effects in these items.

All but one single-item scale had 60% or more participant groups choosing “not at all” at baseline in both the control and intervention groups, indicating a substantial floor effect for these scales (see **Table 3**). The exception was Insomnia where the highest proportion choosing “not at all” was a little over 30% at T2 in both groups, although summing this and the “a little” response options produced a response proportion greater than 60% at baseline in this item also. Inspection of the original distribution of the items (see **Figure 2**) and **Table 2** (dichotomised items) across study time points did not suggest any obvious between group differences or changes in response patterns to these items at the end of the intervention period or the 24-week follow-up, and there was no indication of an arm by time interaction for any of these scales (see **Table 3**).

**Table 3:** Proportion of participants responding “Not at all” to the EORTC QLQ-C30 v 3.0 single-item symptom scales as a function of treatment group for baseline, end of intervention, and post intervention follow-up.

EORTC QLQ-C30 v 3.0	Baseline		12-week		24-week			
	Control (n = 44)	Wearables (n = 43)	Control (n = 41)	Wearables (n = 32)	Control (n = 38)	Wearables (n = 31)	Arm by Time	
Single-item symptom scales	N (%)	N (%)	N (%)	N (%)	N (%)	N (%)	χ^2^(2)	p
Dyspnoea	27 (61)	31 (72)	26 (67)	23 (74)	23 (62)	22 (76)	0.1	0.936
Insomnia	11 (25)	11 (26)	13 (34)	10 (33)	10 (26)	4 (13)	1.2	0.540
Appetite loss	38 (86)	34 (74)	29 (76)	27 (87)	32 (84)	25 (83)	4.0	0.135
Constipation	35 (80)	32 (74)	28 (74)	25 (81)	30 (81)	20 (67)	4.0	0.136
Diarrhoea	36 (82)	37 (86)	33 (87)	26 (84)	29 (78)	21 (70)	2.5	0.290
Financial difficulties	26 (59)	27 (63)	22 (58)	23 (74)	25 (68)	23 (77)	0.9	0.651

N, frequency; % percent of available data (excluding missing values); χ^2^(2)—approximate Wald χ^2^ test for the interaction between arm and time following the mixed model logistic regression.

Missing data: 12-week control—all scales (n = 1) except dyspnoea.

12-week intervention—insomnia (n = 1); 24-week Control—All scales (n = 1) except Insomnia and Appetite loss.

24-week intervention—Dyspnoea (n = 1).

## 4 Discussion

The remotely delivered PPARCS trial significantly increased MVPA in the intervention group (23), and the increase was maintained at follow-up (24). Specifically, the net change in MVPA at follow-up was 52.5 min/week (95% CI 11.0 to 94.0, p = 0.013), with the intervention group showing increased MVPA from T1 to T3 of 67.7 min/week vs. 15.2 min/week among controls (24). Despite these changes, the present analysis indicated that the intervention did not improve HRQoL. The lack of intervention effects may have been due to the relatively small sample size and the notable ceiling and floor effects, with many survivors reporting a relatively high QoL, mostly free of symptoms and with high functioning at baseline, therefore leaving little room for improvement. Nevertheless, there was a clinically meaningful improvement in global QoL in the intervention group at T2 (8.3 points (75 to 83.3)) and was maintained at 24 weeks. In comparison, the control did not improve their global QoL baseline score of 66.7. Despite no evidence of a statistical difference between the groups on global HRQoL, a clinically important change in HRQoL is considered achieved when the improved score matches the criteria for small effects (i.e., a change of 4–10 points), and this is evident for global HRQoL in the intervention group (16). In a meta-analysis of PA interventions in breast cancer, Aune et al. (2022) (16) also found that despite mostly small effect sizes, half of the results were nevertheless considered clinically significant.

There were no statistical between group effects on any of the function scales, although the median difference between groups for physical function was 6.7 points and could be considered a small effect (change of 5 to 15 points) and clinically meaningful (16). Physical function was maintained throughout the trial in the intervention group but decreased in the control group. The symptom scales had response patterns suggestive of significant floor effects. Pain was the only symptom with evidence of an arm by time interaction reflecting the higher pain scores in the control group when collapsed across time points, although pain increased in both groups between end of intervention and follow-up. Increased pain over time has been reported previously (18) and is difficult to explain unless the result of injury from increased PA or weather affecting chronic conditions such as arthritis. These findings are contrary to meta-analytic findings favouring exercise intervention compared with usual care for pain in cancer survivors (standardised mean difference: −0.45) (35).

In general, PA interventions in cancer survivors have resulted in higher global HRQoL and improved physical functioning (16, 36) with weaker effects for emotional and mental functioning (16). To date, there has been limited evidence for the use of mHealth PA interventions to improve HRQoL in cancer survivors. In the few RCTs that have examined the utility of wearable interventions to increase PA and improve HRQoL, most have found no differences in HRQoL between treatment groups (i.e., 37–39), other than for fatigue (measured through the FACIT) favouring the intervention group in a trial using a smart wearable (Garmin Vivofit2) in conjunction with five telephone-based behavioural counselling sessions over 12 weeks (40). There are likely to be several reasons for the ineffectiveness of PA mHealth interventions on HRQoL in cancer survivors, including small sample sizes that are insufficiently powered to detect change, less intensive or formal support, and exercise adherence. For example, Kraemer et al.’s (2022) (41) meta-analysis found no significant effect on QoL for home-based interventions. However, sensitivity analyses found that where intervention adherence was ≥80%, home-based or supervised PA interventions were effective in improving the QoL in CRC patients. The present analysis is one of the first to examine the effects of an entirely distance-based smart wearable intervention, in conjunction with health coaching, on HRQoL in non-metropolitan breast and CRC survivors. We found that PPARCS preserved global health status, with evidence of clinically meaningful improvements in global HRQoL, which is an important outcome.

## 5 Conclusions

Although the PPARCS trial produced a significant increase in MVPA that was maintained at follow-up, the present analysis indicates the intervention did not improve HRQoL. However, findings should be interpreted with caution given the sample size and the high scores for HRQoL observed at baseline limiting room for meaningful change.

### 5.1 Study limitations

Limitations include the small sample size, the relatively high attrition in the intervention group (27.9%), and the low participation rate that likely led to a selection bias and the unintentional recruitment of a sample with relatively high baseline scores for HRQoL contributing to the ceiling and flooring effects observed in the present study.

## Data Availability

The raw data supporting the conclusions of this article will be made available by the authors, without undue reservation.
